# Involvement of mental health professionals in the treatment of tuberous sclerosis complex–associated neuropsychiatric disorders (TAND): results of a multinational European electronic survey

**DOI:** 10.1186/s13023-021-01800-w

**Published:** 2021-05-12

**Authors:** Robert Waltereit, Guillaume Beaure d’Augères, Jasna Jancic, John Chris Kingswood, Maya Koleva, Ruben Marques, Vicente Villanueva, Stéphane Auvin

**Affiliations:** 1 Child and Adolescent Psychiatry and Psychotherapy, University Medical Center Göttingen, Von-Siebold-Str. 5, 37075 Göttingen, Germany; 2 Association Sclérose Tubéreuse De Bourneville, Hindisheim, France; 3 Clinic of Neurology and Psychiatry for Children and Youth, Faculty of Medicine, University of Belgrade, Belgrade, Serbia; 4 St Georges University Medical School, London, UK; 5 University Multiprofile Hospital for Active Treatment in Neurology and Psychiatry “St. Naum”, Sofia, Bulgaria; 6 Novartis Farma S.P.A, Origgio, Italy; 7 Institute of Biomedicine (IBIOMED), University of Leon, León, Spain; 8 Hospital Universitario y Politécnico La Fe, Valencia, Spain; 9 Robert Debré University Hospital, Paris, France

**Keywords:** TAND, TAND checklist, TSC-associated neuropsychiatric disorders, Tuberous sclerosis complex

## Abstract

**Background:**

Tuberous sclerosis complex (TSC) is a rare, genetic, multisystem disorder characterized by the growth of hamartomas in several organs, including the brain, kidneys, heart, eyes, and lungs. Even though over 90% of patients will have some form of TSC-associated neuropsychiatric disorder (TAND), there is an apparent lack of involvement of mental health professionals (MHPs) in the care of patients with TSC. The aim of this study was to determine the current level of TAND awareness in the TSC community and to identify possible barriers to effective multidisciplinary collaboration between MHPs and other healthcare providers (HCPs) in TAND management.

**Methods:**

An electronic survey on current TSC and TAND management was conducted, targeting TSC caregivers/families, psychiatrists, neurologists, TSC specialists, and primary care physicians.

**Results:**

The invitation to participate in the survey was emailed to 659 HCPs and was disseminated through social media channels of patient advocacy groups. The survey was open for 4 months, with 359 responses collected. The majority of participants were TSC caregivers/families (73.3% of all responses). Of the 96 HCPs who participated, most were neurologists (61.5%) or TSC specialists (28.1%). Only 6 psychiatrists and 4 primary care physicians participated. Approximately half of patients have never had a neuropsychiatric assessment, and it was their caregivers/families who initiated the discussion of TAND with their providers. Almost 70% of TSC caregivers/families believed that psychiatric treatment could improve their quality of life. However, 54% of patients had difficulty obtaining psychiatric assessment. In turn, only 21% of HCPs believed that psychiatric therapy would help and 74% were concerned that their patients would be stigmatized by psychiatric referral.

**Conclusions:**

This study focused on European healthcare systems suggests that current care for mental health issues in patients with TSC is inadequate, despite guideline recommendations for regular neuropsychiatric assessments. This appears to be due to a combination of gaps in diagnosis and surveillance, low frequency of psychiatric referrals, insufficient resources, and stigmatization of mental healthcare. There is a pressing need for further initiatives to study and address the mechanisms underlying the mental health treatment gap. The importance of MHP support must be recognized to optimize TSC management.

**Supplementary Information:**

The online version contains supplementary material available at 10.1186/s13023-021-01800-w.

## Introduction

Tuberous sclerosis complex (TSC) is a rare, genetic, multisystem disorder that is estimated to affect 1 in 10,000 live births [1, 2]. TSC is characterized by the growth of hamartomas in several organs, including the brain, kidneys, heart, eyes, and lungs, with a variable clinical presentation depending on the affected organ systems [3, 4]. Neurological manifestations frequently include epilepsy caused by focal malformations during cortical development [5].

In addition to the physical manifestations caused by dysregulated growth control, patients with TSC are also often affected by a spectrum of behavioral, psychiatric, intellectual, academic, neuropsychological, and psychosocial difficulties [5]. TSC-associated neuropsychiatric disorders (TAND) was the term coined by the Tuberous Sclerosis Complex Neuropsychiatry Panel at the 2012 Tuberous Sclerosis Complex International Consensus Conference to encompass these difficulties experienced by patients with TSC [3, 6, 7].

Clinical guidelines for the assessment of these neuropsychiatric disorders were initially published by the TSC Brain/Behaviour Consensus Panel in 2005 [8]. Healthcare providers (HCPs) from around the world with expertise in managing TSC convened in June 2012 to update the existing guidelines for the diagnosis, surveillance, and management of TSC. The consensus that was reached as a result of the work before, during, and after that conference was published in the October 2013 edition of *Pediatric Neurology* [1, 6].

The TAND checklist is a clinical tool that can be used to identify neuropsychiatric areas that require further evaluation or treatment [3, 5, 9]. The checklist was developed by the Tuberous Sclerosis Complex Neuropsychiatry Panel to provide a simple framework for a conversation between the clinician and the family or individual with TSC regarding TAND [3, 10].

Current guidelines [6, 8] recommend regular assessments of cognitive development and behaviors in patients with TSC to establish a baseline for evaluating any changes in developmental trajectory. However, a survey conducted among members of the UK Tuberous Sclerosis Association in 2010 showed that only 18% of all families received any of the evaluations recommended in the 2005 guidelines [3]. Given that more than 90% of patients with TSC are likely to have some neuropsychiatric problems in their lifetime, the gap between the clinical need and services provided is in excess of 70% [3, 10]. This difference reflects the global findings of treatment gaps in mental health, where an estimated 70–80% of individuals with psychiatric disorders do not receive treatment [3, 11].

Mental health professionals (MHPs) involved in the treatment of TAND include psychiatrists, clinical psychologists, speech and language therapists, occupational therapists, and many others. Some of these professions have additional sub-specializations for child and adolescent patients versus adult patients. A consultant psychiatrist often leads the multidisciplinary team of MHPs and acts as the group’s liaison with other HCPs [12, 13]. Therefore, psychiatrists are well acquainted with issues related to healthcare resources and the stigmatization of mental disorders. For the purposes of this study, we chose to focus on psychiatrists to represent the mental health multidisciplinary team and did not distinguish between child and adolescent psychiatrists versus general psychiatrists.

In a meeting of an international panel of TSC experts in Europe in 2019, five hypotheses were proposed to account for the gap in mental health treatment in TSC (Table 1). Here, we report the outcomes of an online survey that was formulated and conducted to explore these hypotheses, to determine whether there is a lack of involvement of MHPs in the treatment of TAND, and to identify any barriers to MHP engagement in TSC screening, diagnosis, and treatment.

**Table 1:** Hypotheses for the gap in mental health treatment in TSC

1. Lack of resources in psychiatry
2. Lack of resources for multidisciplinary team interactions
3. Lack of knowledge about psychiatry among general HCPs and/or TSC caregivers/families, leading to reluctance in referring patients to psychiatry
4. Lack of knowledge about non-psychiatric healthcare among psychiatrists, resulting in diminished confidence among psychiatrists in treating patients with TSC
5. Stigmatization of psychiatry among non-psychiatric HCPs and/or TSC caregivers/families

*HCPs* healthcare providers, *TSC* tuberous sclerosis complex

## Methods

### European TSC expert meeting

This study aimed to determine whether there was a lack of psychiatric services involvement in the treatment of TAND by European healthcare services, and to identify barriers that affect psychiatric involvement and multidisciplinary collaboration. The meeting of the EXchanging PERspectives on TSC (EXPERT) Steering Committee, comprising seven European TSC experts, was held on February 26, 2019, in Paris, France. At the meeting, the Steering Committee members developed five hypotheses. They decided to study the research question using an electronic survey, conducted partially among TSC caregivers/families, and partially among four groups of HCPs: TSC specialists (defined as working in a TSC clinic), neurologists/child neurologists, psychiatrists/child psychiatrists, and physicians with limited experience in treating patients with TSC. Although mental healthcare services are provided by different professions, the expert group decided to focus on psychiatrists because they are the key MHPs in most European medical healthcare systems.

### Development of questionnaires

The EXPERT Steering Committee developed two core questionnaires to test these hypotheses: one for TSC caregivers/families and one for HCPs. Each subgroup of HCPs was presented with a slightly different version of the questionnaire. Each questionnaire consisted of 8 to 10 questions, depending on its target group (see Additional files 1–5: Tables S1–S5). Survey questions were refined to cover all five hypotheses proposed during the Steering Committee meeting; questions were based on the clinical experience of the members and their views on the treatment of TSC patients and potential barriers to mental healthcare in European healthcare systems. A guiding principle of the meeting was to allow novel views and ideas to generate unorthodox viewpoints on established European healthcare systems. As the study had an exploratory nature, questions were not derived from existing psychometric tools, but rather formulated from open discussion between experts. In addition, each questionnaire was the product of an individual discussion among all members of the Steering Committee, with the aim of formulating the best questions and selection of possible answers for the respective target group. Questions focused on the conduction of TAND assessments, referrals to psychiatric services, potential barriers to effective management of TSC, and the experience of patients, families, and caregivers. The selected questions were translated into Bulgarian, French, German, Italian, Serbian, and Spanish.

### Survey methodology

The survey was distributed to five recipient groups: TSC caregivers/families, TSC specialists, neurologists/child neurologists, psychiatrists/child psychiatrists, and physicians with limited experience with TSC. HCPs were invited to participate via email, whereas TSC caregivers/families were notified through social media platforms. Email addresses were collected from the Steering Committee’s personal professional networks, with an aim to include similar proportions of target recipients per specialty. The survey was conducted online through SurveyMonkey^®^.

## Results

### Demographics

The invitation to participate in the survey was sent to 659 HCP email addresses submitted by the members of the EXPERT Steering Committee and was disseminated through the social media channels of patient advocacy groups. There were 359 respondents, most of whom were TSC caregivers/families (*n* = 263; 73.3%). A total of 96 responses were from HCPs (15% response rate), comprising 26.7% (*n* = 96) of all survey responses (Table 2). Of the 96 HCPs who responded, 59 were neurologists (61.5%) and six were psychiatrists (6.3%). Of the remaining HCP respondents, 27 were TSC specialists (28.1%) and four were HCPs with limited TSC knowledge (4.2%).

**Table 2:** Demographics of survey participants

Characteristics, n (%)	HCPs contacted via email (specialty verified, *N* = 659)	Respondents (*N* = 359)
TSC caregivers/families	N/A^a^	263
HCPs	659	96 (15% HCP response rate)
TSC specialists	9	27^b^
Neurologists (including neuropsychiatrists)^c^	194	59
Psychiatrists	154	6
HCPs with limited experience with TSC^d^	104	4
Other specialties^e^	34	0
Specialty not specified	164	0

*HCP* healthcare provider, *TSC* tuberous sclerosis complex

^a^TSC caregivers/families were invited to participate in the survey via social media channels and the total number of people reached via social media platforms cannot be ascertained

^b^The most likely reason for the larger recorded number of TSC specialists who responded to the survey than those contacted via email was that it was difficult to confirm an HCP’s participation in a multidisciplinary TSC clinic based on contact details alone. However, HCPs were directed to specific questionnaires based on responses to initial questions regarding their clinical practice, so that it was possible to identify which participants were considered TSC specialists

^c^In some European countries, neuropsychiatrists are physicians with a double qualification in neurology and in psychiatry. For the purposes of our study, we included neuropsychiatrists in the group of neurologists

^d^HCPs with limited experience with TSC refers mainly to primary care physicians

^e^Other specialties included nephrology (13), pulmonology (4), laser medicine (3), genetics (4), oncology and hematology (3), cardiology (1), radiology (1), urology (4), and endocrinology (1)

The countries with the highest total number of respondents were Italy (*n* = 65, 18.1%), Germany (*n* = 63, 17.5%), and France (*n* = 61, 17%). HCPs from 25 different countries responded, with the highest proportion from Germany (*n* = 15, 15.6%); of the psychiatrists who responded, three were from Serbia, two were from Germany, and one was from Bulgaria (Additional file 6: Fig. S1). TSC caregivers/families from 23 different countries responded; most of them were from Italy (*n* = 58, 22.1%), France (*n* = 57, 21.7%), and Germany (*n* = 48, 18.3%) (Additional file 7: Fig. S2).

### TAND assessments

**Fig. 1:**
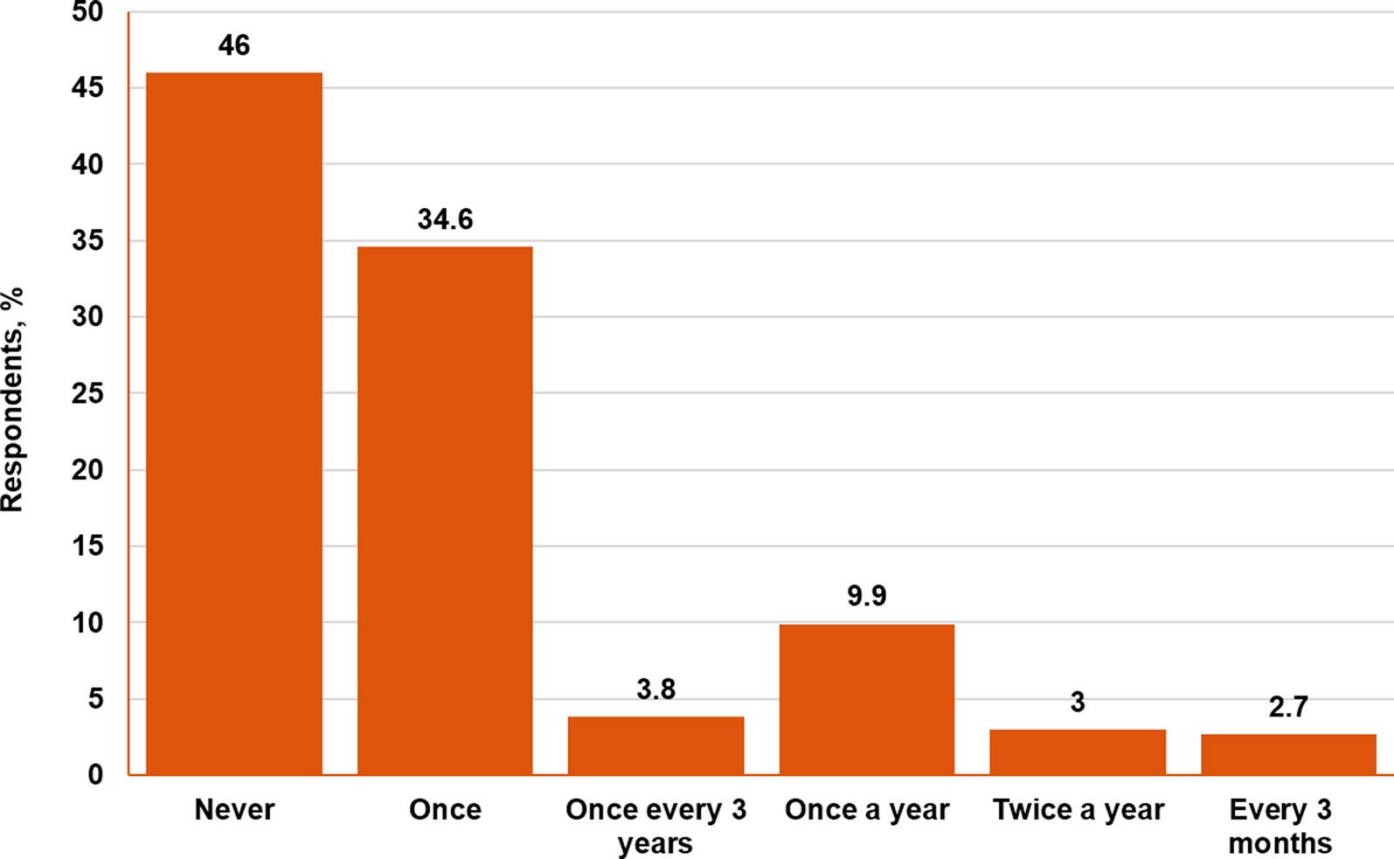
Frequency of neuropsychiatric assessment according to TSC caregivers/families

**Fig. 2:**
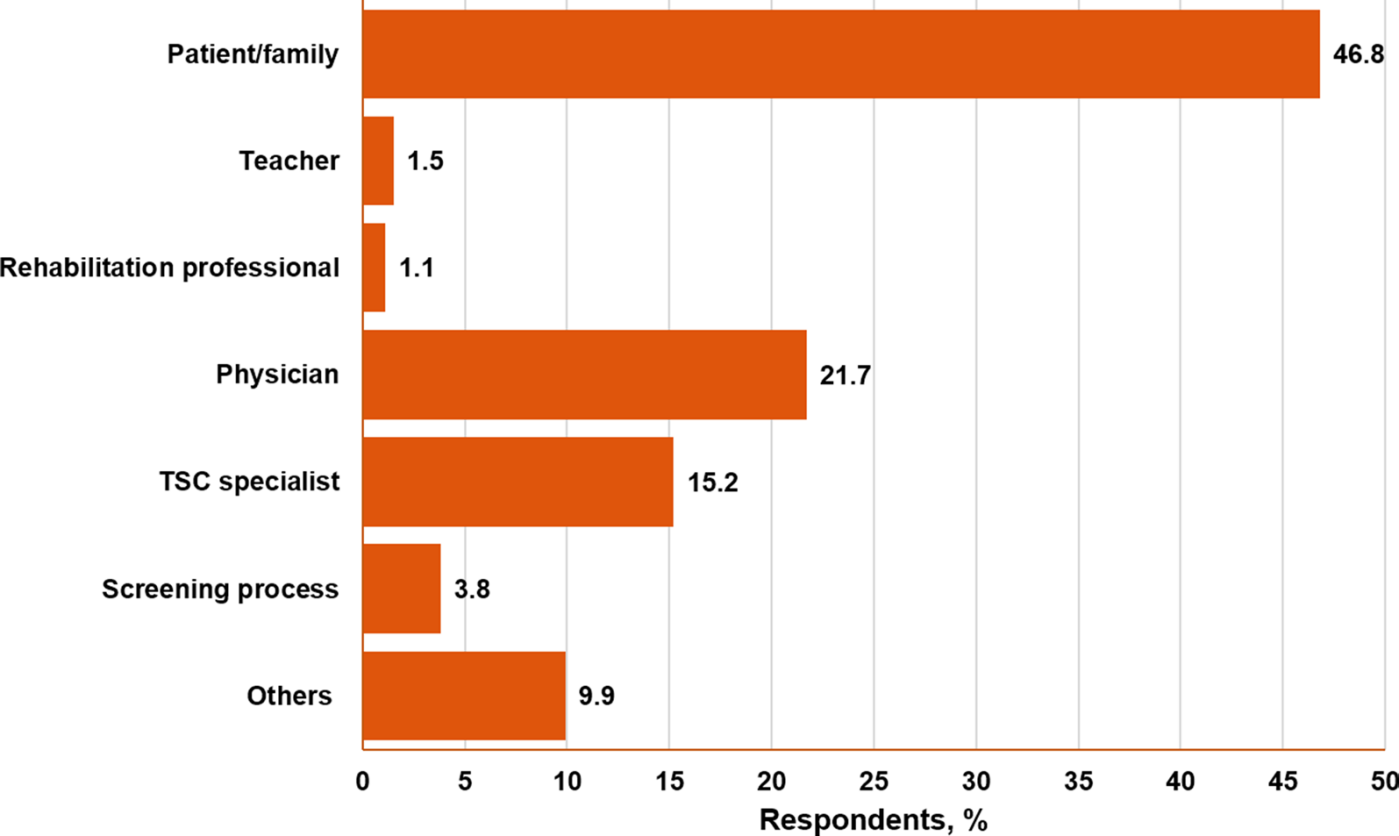
Source of TSC caregivers/families’ awareness of TAND. *TAND* tuberous sclerosis complex–associated neuropsychiatric disorders; *TSC* tuberous sclerosis complex

A neuropsychiatric assessment of TAND symptoms in a TSC patient by an HCP takes the form of a clinical interview, for which the TAND checklist was developed as a supporting tool [3]. According to the survey, 46% of patients (121/263) never had a neuropsychiatric assessment or filled out a TAND checklist, whereas 9.9% of patients (26/263) were screened for TAND once a year (Fig. 1). In nearly 50% of cases (123/263), TSC caregivers/families were the ones who raised the topic of TAND with their HCP. Only 3.8% of TSC caregivers/families (10/263) learned about TAND from their providers during the screening process for TSC (Fig. 2).

**Fig. 3:**
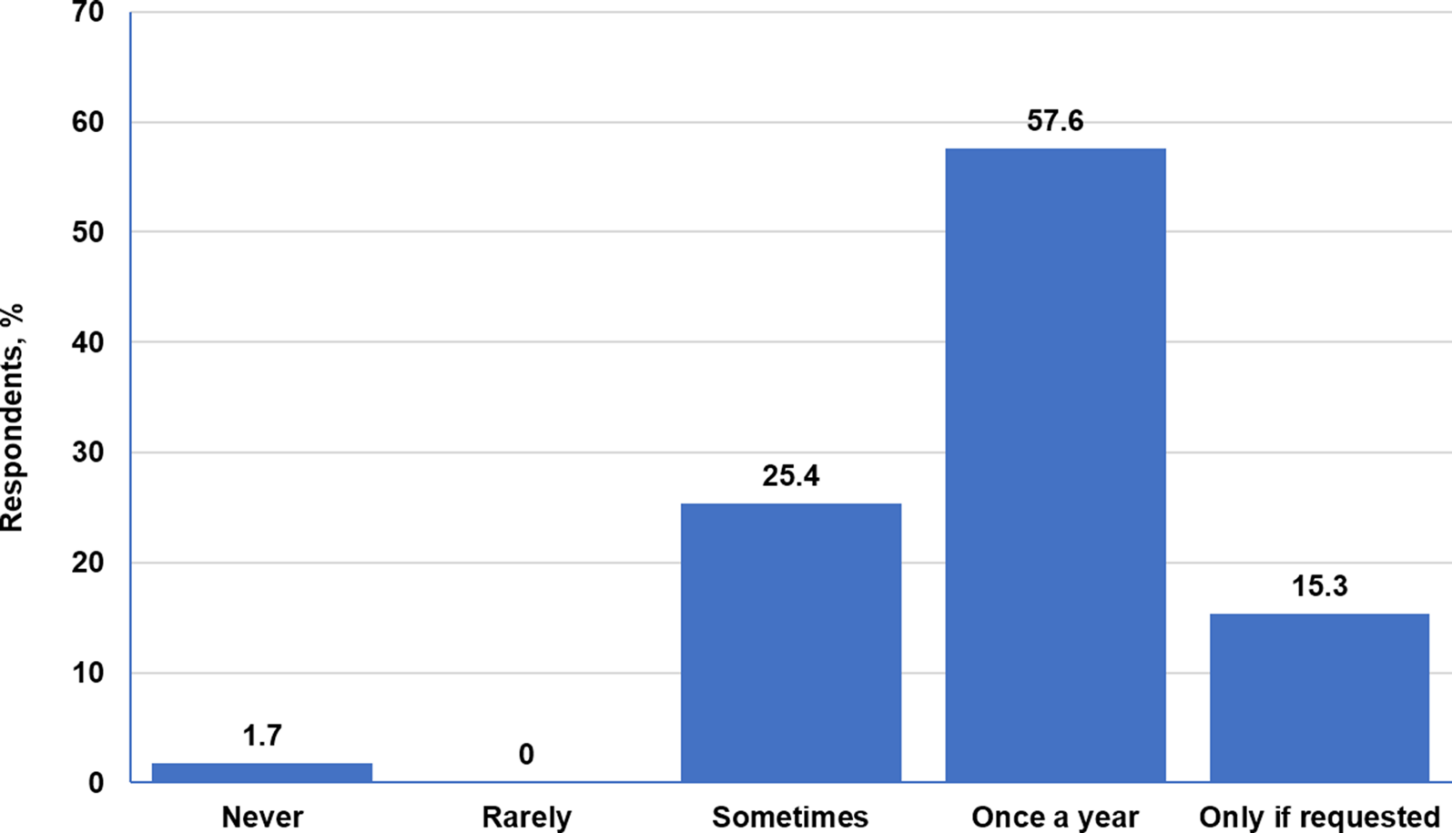
Frequency of neuropsychiatric assessments by neurologists

**Fig. 4:**
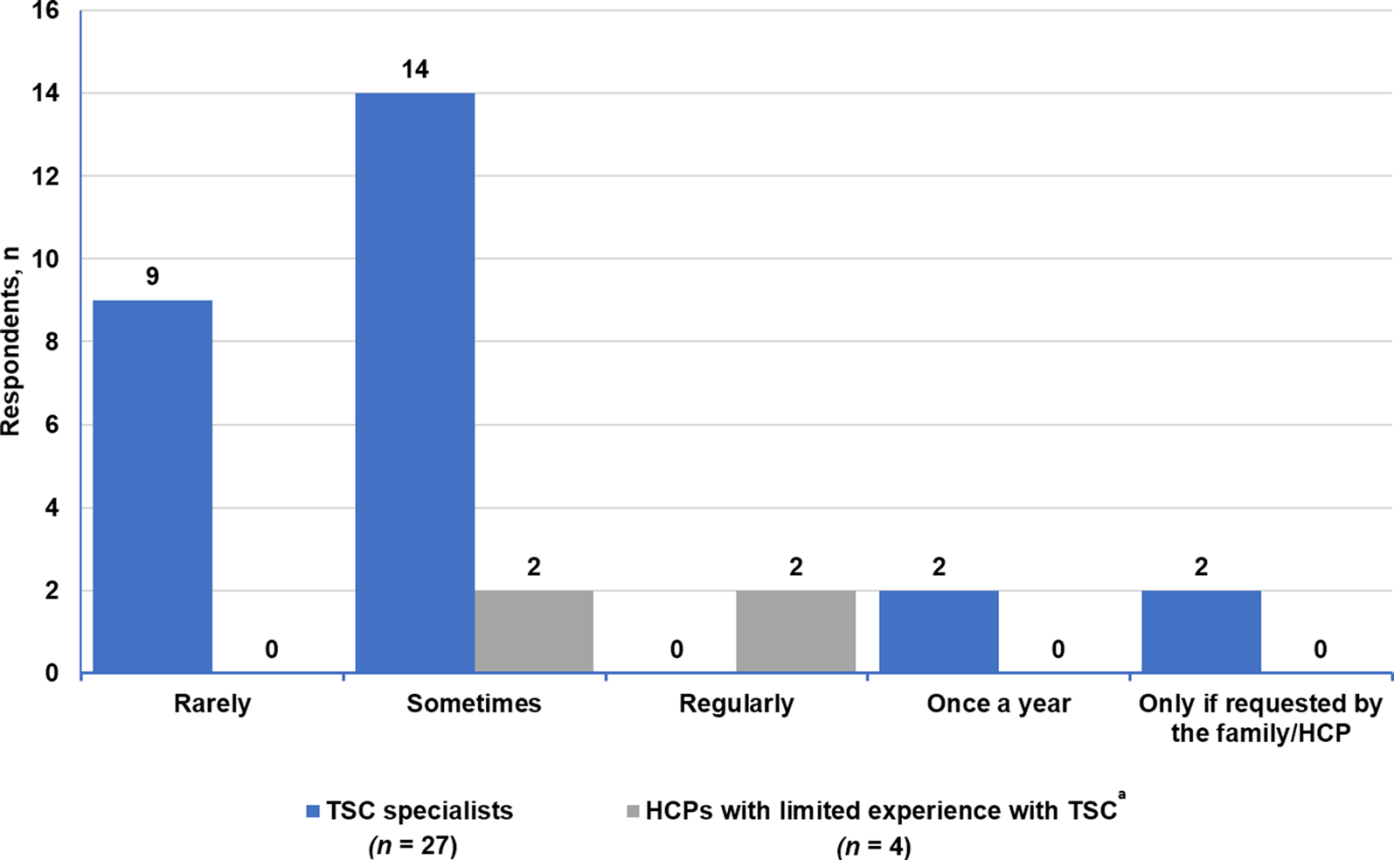
Frequency of referral to psychiatry by TSC specialists and HCPs with limited experience with TSC. ^a^*HCPs* healthcare providers, *TSC* tuberous sclerosis complex. ^a^HCPs with limited experience with TSC refer mainly to primary care physicians

More than half of neurologists (57.6%, 34/59) screened their patients with TSC for psychiatric or neurocognitive issues once a year, whereas 16.9% of neurologists (10/59) answered “never” or “only upon request” when asked about the frequency of TAND screening (Fig. 3). None of the HCPs with limited experience with TSC screened their patients for neurocognitive problems (Fig. 4); they were most likely to perform skin and eye examinations (Additional file 8: Fig. S3).

### Psychiatric referrals

**Fig. 5:**
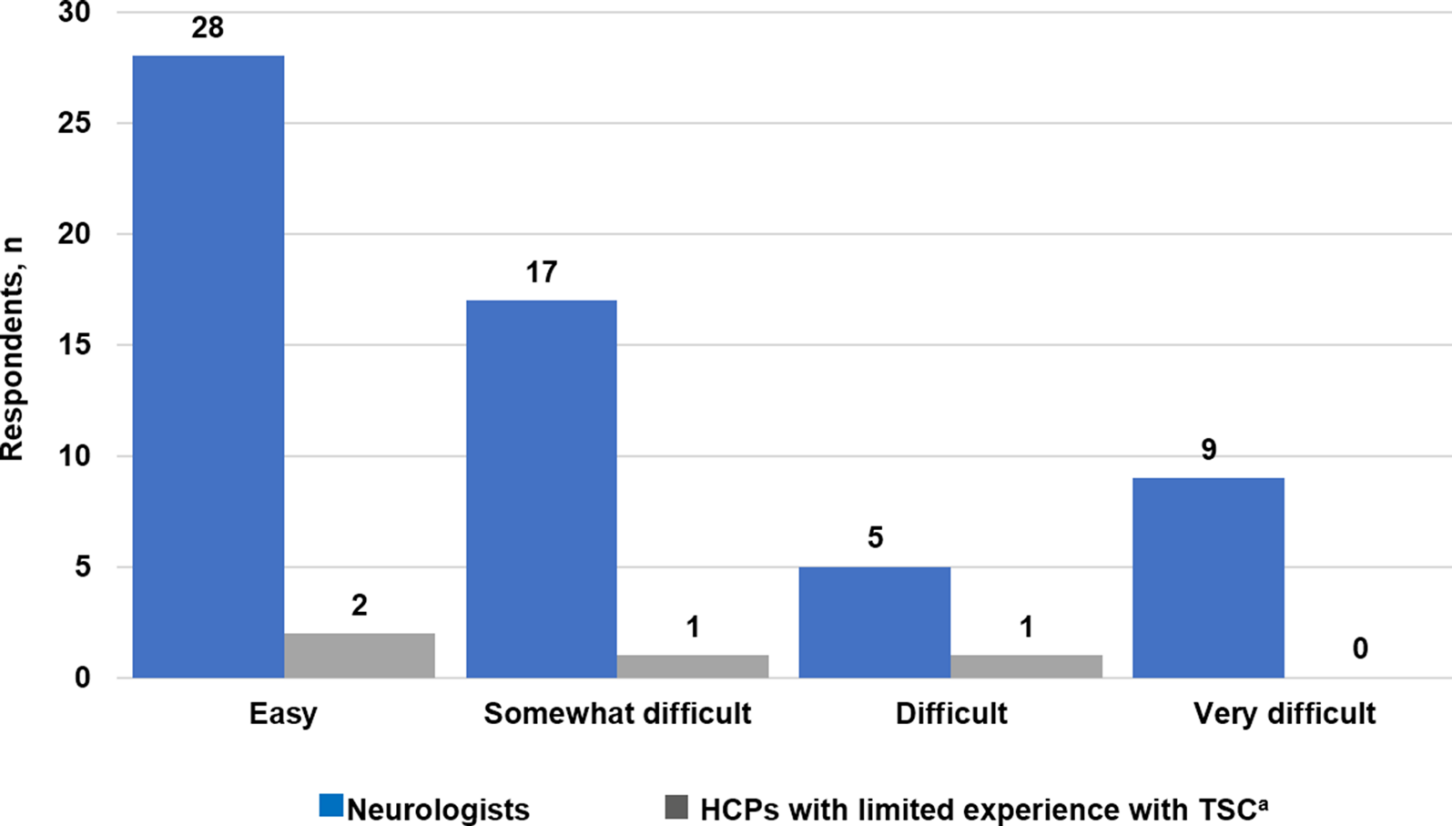
Ease of referring to psychiatry according to neurologists and HCPs with limited experience with TSC. ^a^*HCPs* healthcare providers, *TSC* tuberous sclerosis complex. ^a^HCPs with limited experience with TSC refer mainly to primary care physicians

Among the TSC specialists, 85.2% (23/27) said that they sometimes or rarely referred their patients to psychiatry (Fig. 4). TSC specialists were generally able to manage behavioral and cognitive impairments but referred the following to psychiatrists: self-harming behavior, aggression, sudden rage, attention deficit hyperactivity disorder, obsessive–compulsive disorder, and autism (Additional file 9: Fig. S4). Almost half of neurologists (47.5%, 28/59) generally found it easy to refer a patient with TSC to a psychiatrist (Fig. 5). Among the four HCPs with limited TSC experience, only two respondents regularly referred their patients with TSC to psychiatry (Fig. 4). Only three of the six psychiatrist respondents (50%) said that there were enough resources in psychiatry in their center to handle referrals from other specialties.

**Fig. 6:**
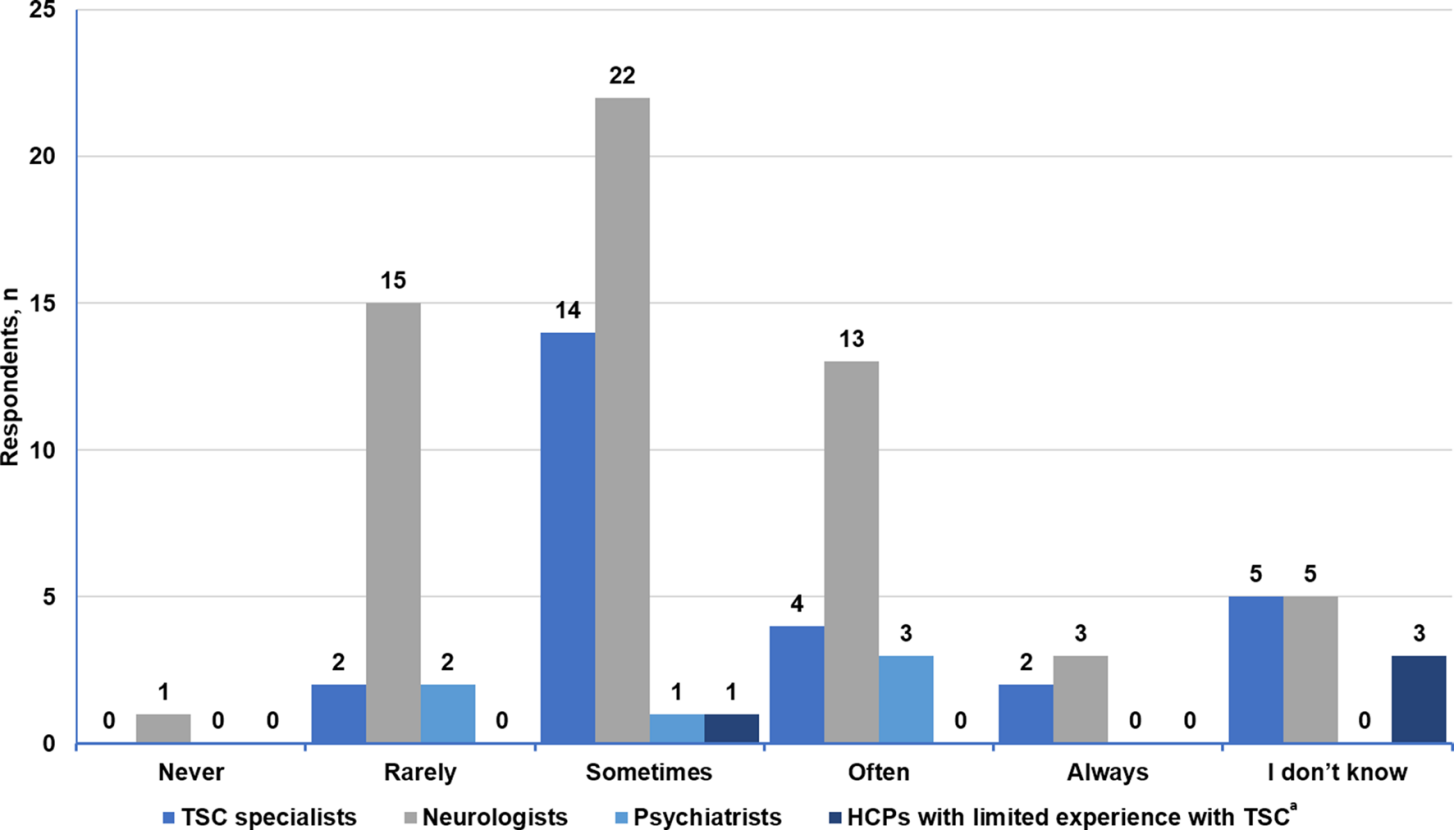
Perceived effectiveness of psychiatric therapy on TAND according to HCPs.^a^ *HCPs* healthcare providers, *TAND* tuberous sclerosis complex–associated neuropsychiatric disorders; *TSC* tuberous sclerosis complex. ^a^HCPs with limited experience with TSC refer mainly to primary care physicians

**Fig. 7:**
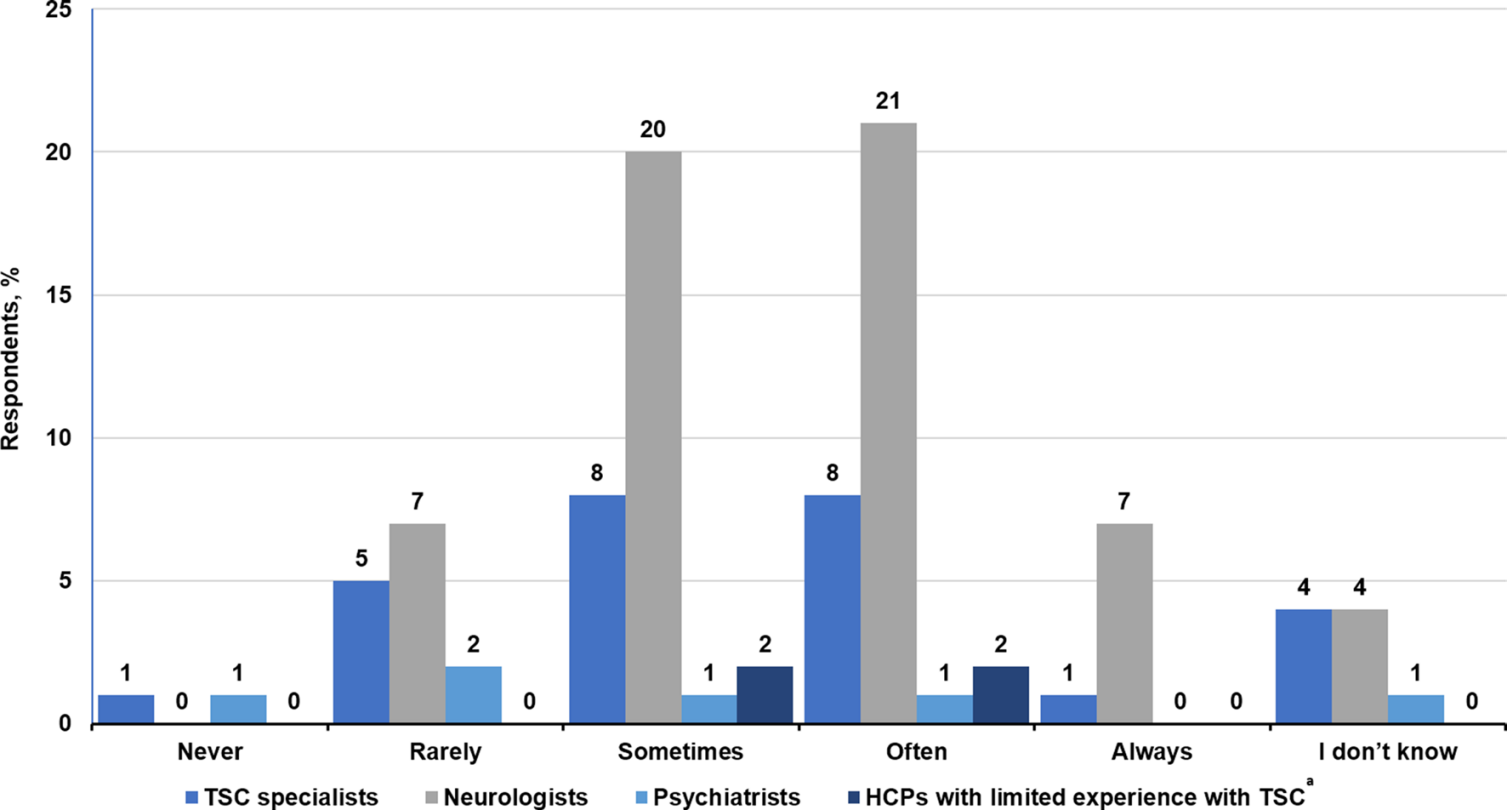
Perceived stigmatization of patients according to HCPs. *HCPs* healthcare providers, *TSC* tuberous sclerosis complex. ^a^HCPs with limited experience with TSC refer mainly to primary care physicians

More HCPs (39.6%, 38/96) thought that standard psychiatric therapy only sometimes worked for patients with TSC, as opposed to 20.8% of HCPs (20/96) who believed that standard psychiatric therapy often worked for patients with TSC (Fig. 6). The majority of HCPs surveyed (74%, 71/96) believed that their patients would feel stigmatized when referred to psychiatric services (Fig. 7).

### Potential barriers to effective management of TSC

**Fig. 8:**
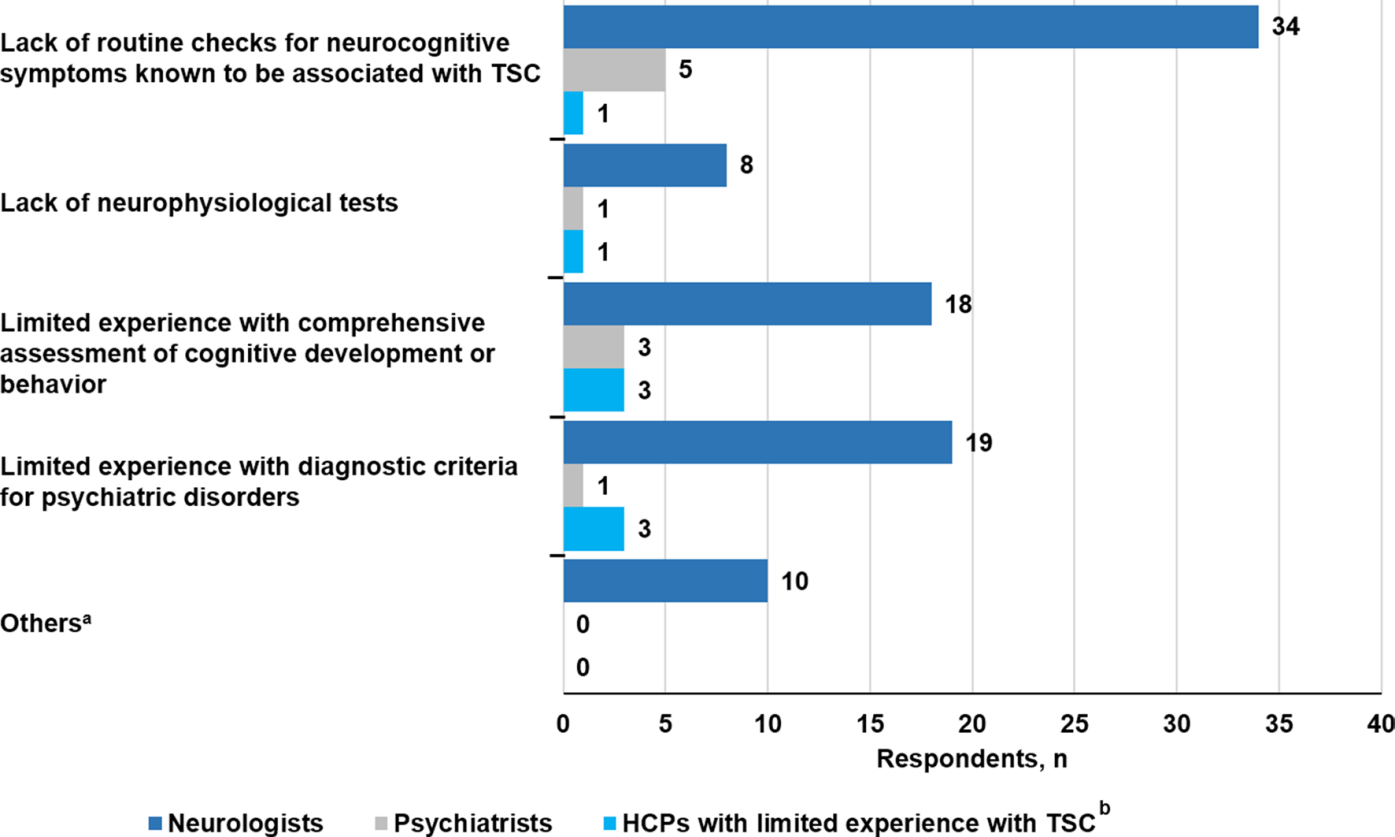
Barriers to effective TAND assessment according to HCPs. *HCP* healthcare provider, *TAND* tuberous sclerosis complex–associated neuropsychiatric disorders, *TSC* tuberous sclerosis complex. ^a^Answers supplied by respondents under the “Others” field were: (1) No barrier in our institution/country (2 responses); (2) Health economic barriers, lack of refinancing, etc.; (3) A lack of interest and experience on the part of the practitioner despite current data and knowledge of the need for routine examinations and standardized tests; (4) Lack of funding for outpatient examinations; (5) Lack of time and changing caregivers with different expressiveness; (6) I ask about sleep disorders or relational disorders, but forget to distribute/address TAND regularly; (7) Difficulty obtaining support in psychiatry; (8) Lack of time in consultations, not all centers have access to a pediatric neuropsychologist; (9) Generally time and capacity. ^b^HCPs with limited experience with TSC refer mainly to primary care physicians

**Fig. 9:**
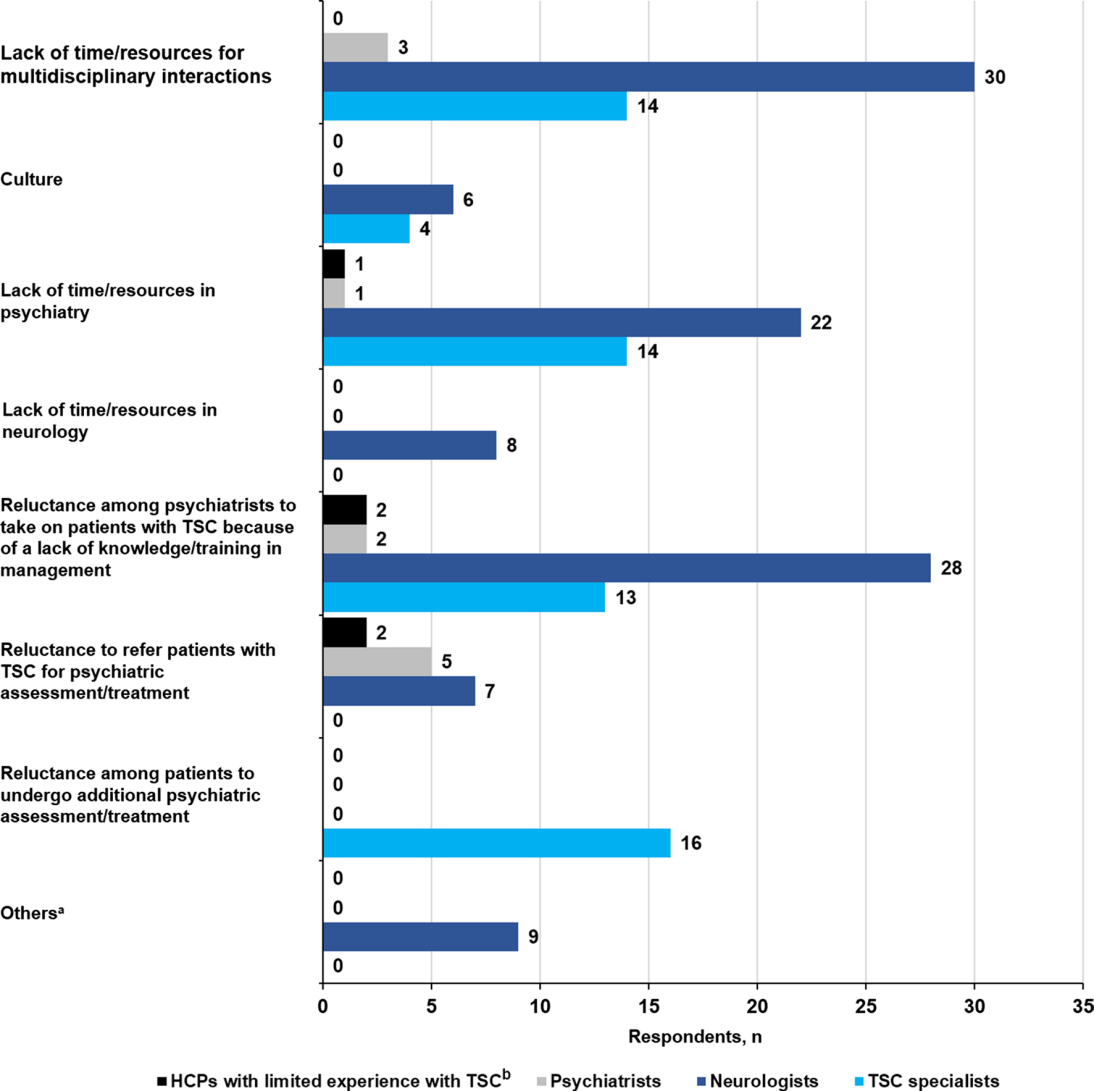
Barriers to effective collaboration according to HCPs. *HCPs* healthcare providers, *TSC* tuberous sclerosis complex. ^a^Answers supplied by respondents under the “Others” field were: (1) None/no barriers (5 responses); (2) Psychiatric reluctance to receive patients with "organic" pathology; (3) Clinic operates as MDT with attending psychologists. Child psychiatrists are available for consultation and advice but do not attend the dedicated TSC clinic; (4) Can only comment on children and young people; (5) Sectoral separation. ^b^HCPs with limited experience with TSC refer mainly to primary care physicians

The top three barriers to effective assessment of neuropsychiatric difficulties in patients with TSC cited by HCPs were: (1) a lack of routine checks for neuropsychiatric symptoms known to be associated with TSC (41.7%, 40/96); (2) limited experience with comprehensive assessment of cognitive development or behavior (25.0%, 24/96); and (3) limited experience with diagnostic criteria for psychiatric disorders (24.0%, 23/96) (Fig. 8). The top three barriers to effective collaboration cited by HCPs were: (1) a lack of time or resources for multidisciplinary interactions (49%, 47/96); (2) reluctance among psychiatrists to take on patients with TSC because of a lack of knowledge or training in management (46.9%, 45/96); and (3) a lack of time or resources in psychiatry (39.6%, 38/96) (Fig. 9).

### Experience of patients, families, and caregivers

**Fig. 10:**
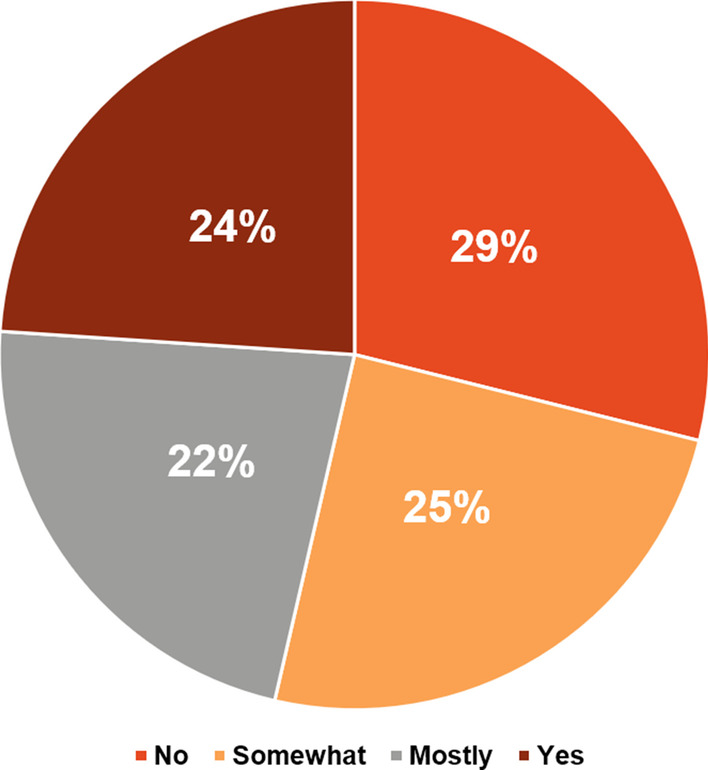
Perceived adequacy of management of neuropsychiatric requirements of TSC caregivers/families. *TSC* tuberous sclerosis complex

**Fig. 11:**
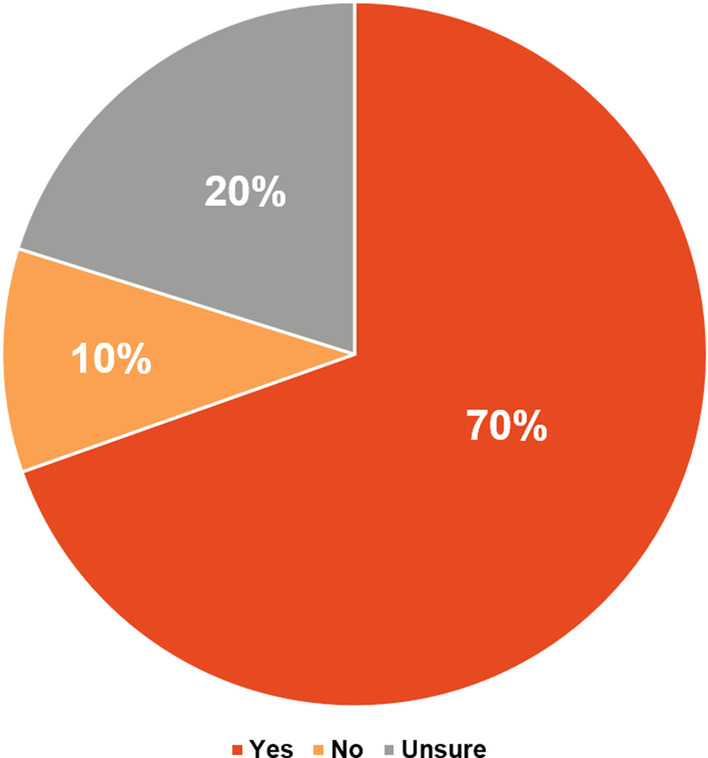
Potential improvement in quality of life with psychotherapy according to TSC caregivers/families. *TSC* tuberous sclerosis complex

**Fig. 12:**
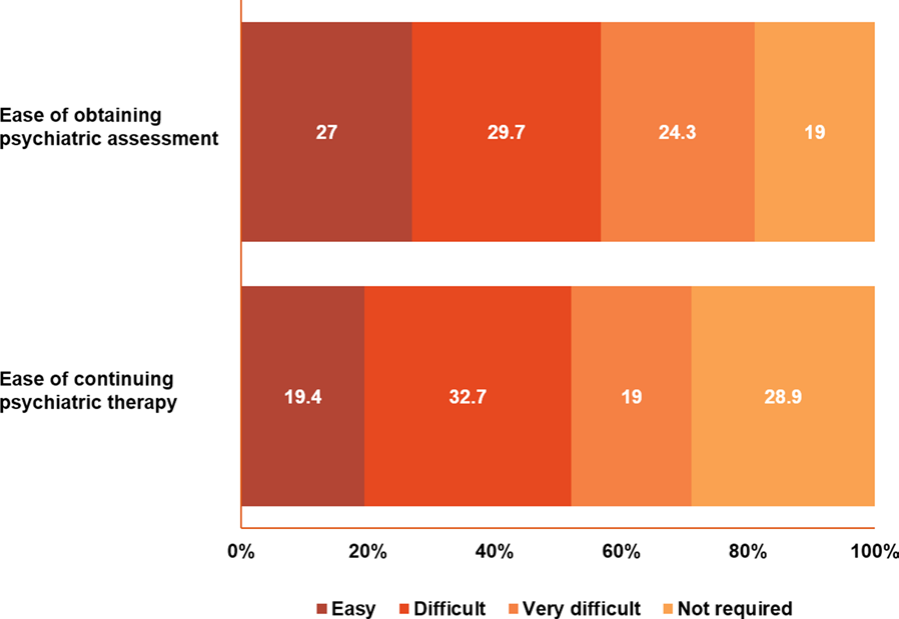
Access to psychiatric resources according to TSC caregivers/families. *TSC* tuberous sclerosis complex

**Fig. 13:**
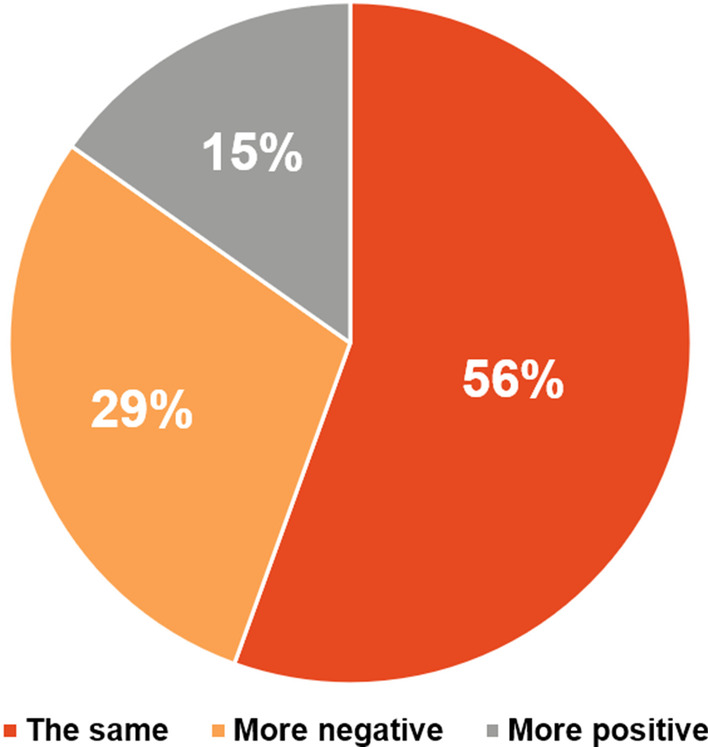
Perception of TSC caregivers/families when referred to psychiatry versus other health services. *TSC* tuberous sclerosis complex

Despite the fact that 46% of patients (121/263) never had a neuropsychiatric assessment or filled out a TAND checklist, only about one-third (29%, 76/263) of TSC caregivers/families felt that their family member’s neuropsychiatric requirements were not appropriately managed by their HCP (Fig. 10). Almost 70% of TSC caregivers/families (183/263) believed that psychiatric treatment could improve quality of life (Fig. 11). However, more than half (54%, 142/263) of TSC caregivers/families found it difficult or very difficult to obtain a psychiatric assessment. Of the patients who had initiated psychiatric therapy, 51.7% (136/263) of TSC caregivers/families found it “difficult to very difficult” to continue psychiatric therapy (Fig. 12). In contrast to the stigma perceived by HCPs of referral to psychiatric services (Fig. 7), most TSC caregivers/families (70.7%, 186/263) did not feel more negative after being referred to a psychiatrist than when they were referred for other healthcare assessments (Fig. 13).

**Fig. 14:**
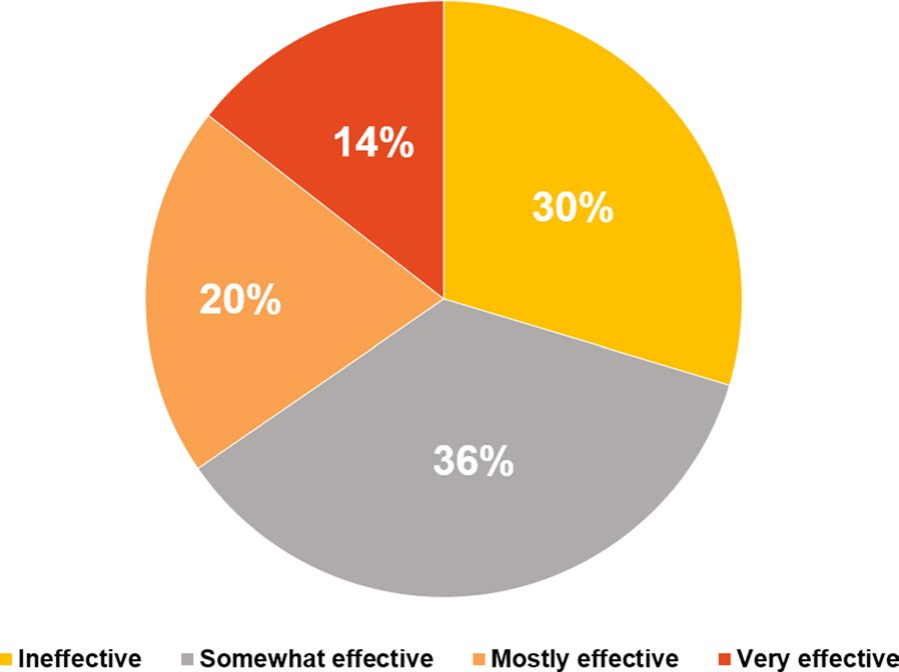
Perception of TSC caregivers/families of the effectiveness of collaboration between their HCPs. *HCPs* healthcare providers, *TSC *tuberous sclerosis complex

A larger proportion of TSC caregivers/families found the collaboration between their doctors to be ineffective to somewhat effective (65.4%, 172/263), whereas only 34.6% of TSC caregivers/families (91/263) found multidisciplinary collaboration to be mostly or very effective (Fig. 14).

## Discussion

This online survey targeting the European TSC community was the first one to study the role and involvement of MHPs in the treatment of TAND. It demonstrates the substantial lack of awareness of TAND among both HCPs and TSC caregivers/families. The results of this study support the main hypothesis raised by the EXPERT Steering Committee, which was that the gap in mental health treatment for TSC is a result of low involvement among MHPs in the management of patients with TSC. In the following, we will discuss our main findings before analyzing them in perspective against the initial five hypotheses.

Despite the efforts to reach as many individuals involved in the care of patients with TSC as possible with this survey, there was a low response rate from HCPs in general. Most HCP respondents were either neurologists or TSC specialists; only six psychiatrists and four HCPs with limited experience of TSC participated in the survey. This observation could reflect the lack of exposure to, and experience with, TSC, given that it is a rare disease entity with highly variable physical and neuropsychiatric manifestations. Despite these numbers, we were able to solicit responses from HCPs from 25 different countries owing to the electronic format of the survey.

A relatively large number of responses were received from TSC caregivers/families. To target this group, the survey link was disseminated through social media platforms. Recent studies on patient recruitment strategies for studies of rare diseases show that online advertising through social media channels is an effective user-targeted recruitment tool [14–16]. Our survey was able to reach a substantial number of TSC caregivers/families from 23 different countries through these platforms, in line with the results from patient engagement initiatives of other groups.

The low number of patients who have received neuropsychiatric assessment confirms the findings of a previous study from the United Kingdom [3] that there is large gap in the neuropsychiatric assessment and treatment of patients with TSC throughout Europe. Lack of awareness of TAND and the TAND checklist among HCPs were found to be important factors contributing to this treatment gap in the care of patients with TSC [17].

One possible reason for the low involvement of MHPs in the management of TSC and TAND could be a *lack of resources* in psychiatry, which was cited by HCPs as one of the top three barriers to effective multidisciplinary collaboration. Of the six psychiatrists who participated in our survey, only three of them believed that there were enough psychiatric resources in the centers where they practice. However, given these low numbers, these insights can only be counted as personal opinions. According to World Health Organization (WHO) data, the WHO European region has the largest mental health workforce, with 50 mental health workers available per 100,000 population. However, this number includes psychiatrists, nurses, social workers, and speech therapists [18]; the median number of psychiatrists and child psychiatrists in the European Union is much lower, at 10 per 100,000 population [19], with considerable variation among the different European countries [18]. Nevertheless, a general lack of resources in Europe does not appear to be the primary reason for low psychiatrist engagement. However, the psychiatrists’ general impression that TSC patients were more “difficult to manage” than those with idiopathic mental disorders may produce a “relative” lack of resources in the sense of having fewer psychiatrists who are willing to handle TSC patients.

The strikingly low proportion of psychiatrists who participated in our survey could partly be due to a limitation of psychiatric resources, as mentioned previously. Limited availability could result in *the reluctance of psychiatrists* to actively enroll patients with TSC into their treatment facilities, given that patients with TSC are usually more complex and difficult to treat than most psychiatric patients without a multisystem genetic syndrome. The comorbidity of neurologic and physical diseases could discourage psychiatrists in accepting referrals of TSC patients, further contributing to the knowledge gap with regard to TAND. This interpretation is in line with the historical treatment gap in psychiatry for patients with intellectual disability, which is also common in TSC patients [20]. The combination of these factors may account for the low involvement of psychiatrists in the care of TSC patients. However, these findings warrant further exploration because our study elicited very few responses from psychiatrists, despite our extensive efforts to include a large sample of psychiatrists in the survey.

The *stigma* surrounding mental health disorders is another possible barrier to the engagement of psychiatric services in the care of TSC patients [21]. Sixty-three percent of physicians thought that patients or their family members often or sometimes felt stigmatized when referred to psychiatry. However, the majority (70.7%) of TSC caregivers/families did not feel more negative after being referred to a psychiatrist than when they were referred for other healthcare assessments. As with other mental health disorders, there appears to be a need for anti-stigma campaigns involving both HCPs and patients’ families to establish accessible and effective mental health services for patients with TSC [18, 21].

Most of the specialists who work in TSC clinics only sometimes, or even rarely, refer their patients to psychiatry for co-management, whereas two of the four HCPs with limited TSC experience regularly refer their patients to psychiatry. These findings could highlight a need for the adequate training of HCPs in the identification of patients who require psychiatric referral, especially for HCPs in the primary care system, which is often the first point of access for most people with mental health problems [21].

A large proportion of TSC caregivers/families are not satisfied with the collaboration between their doctors, with only 34.6% of TSC caregivers/families stating that they found multidisciplinary care to be mostly effective or very effective. When HCPs were asked about the possible *barriers for effective collaborations*, almost half of all HCP responders cited the lack of time or resources as the main reason. The second most reported barrier to effective collaboration was the reluctance among psychiatrists to accept referrals of patients with TSC because of a lack of knowledge or training in the management of TSC and TAND. The low number of psychiatrists who responded to the survey may reflect a certain level of doubt in considering TAND as an area of need for psychiatric services, as we have discussed above. One of the HCP respondents further elaborated that there is some reluctance in the psychiatric community to receive patients with “organic” pathology.

We will now analyze our main findings in perspective against the *initial five hypotheses*. First, lack of resources in psychiatry: The findings from our survey support a lack of resources; however, they cannot estimate its extent. Second, lack of resources for multidisciplinary team interactions: The findings from the survey indicate a lack of resources but, again, we cannot measure their extent. Third, lack of knowledge about psychiatry among non-psychiatric HCPs and/or TSC caregivers/families, leading to reluctance in referring patients to psychiatry: The relatively high rate of psychiatry stigmatization among HCPs, compared with patients’ families, suggests a gap in knowledge about mental healthcare. Fourth, lack of knowledge about non-psychiatric healthcare among psychiatrists, resulting in a lack of confidence among psychiatrists who are treating patients with TSC: This can be backed up by the very low rate of psychiatrists who participated in our survey. Fifth, stigmatization of psychiatry among non-psychiatric HCPs and/or TSC caregivers/families: A surprising finding of our survey was that stigmatization of psychiatry appeared to be higher among HCPs compared with families. This could be an especially important finding and deserves to be considered in future strategies to improve the care of TAND.

Based on the findings of the survey, the EXPERT Steering Committee discussed *potential strategies* to address the mental health treatment gap. One of these strategies could be to engage the global TSC community and regional TSC associations to effectively increase the number of people reached by awareness campaigns and other initiatives. Patients, families, and HCPs could all benefit from activities such as TAND awareness campaigns and educational programs on the diagnosis and evolving therapeutic landscape of TAND; they could also serve as a starting point for eradicating the stigma surrounding mental health.

Recently, the *TANDem research consortium* (https://tandconsortium.org), a working group of international TAND experts, has started such an approach with a specific working program. The focus is not on the direct improvement of structures in healthcare systems, but rather on empowering families of TSC patients by: (1) developing a self-completion, quantified TAND checklist, which is built into a smartphone application; (2) generating consensus guidelines for the treatment of TAND, which are built into the TAND smartphone application; and (3) engaging in a range of networking activities to help empower broader communities with the knowledge and tools to better understand TAND [22].

Another approach could be development of an improved *strategy for multidisciplinary teams* in the treatment of TSC, with well-defined roles for each member of the team, which would likely improve the care of patients with TAND [6]. Ideally, one main provider would lead the team and be responsible for areas or issues that fall outside the specific obligations and responsibilities of other team members. A guidelines-based approach in the overall management would ensure that care is optimized throughout the key developmental stages in the patient’s life, and that regular and timely neuropsychiatric assessments are performed with the help of the TAND checklist [6]. Once a patient is referred to psychiatry or MHPs in general, it would be essential to build a relationship of trust between the patient and the psychiatrist or MHP to maximize the potential for positive results.

The *main limitation* of our study is the small sample size of 359 respondents—over 70% of whom were TSC caregivers/families. There were only six psychiatrists and four HCPs with limited experience with TSC who participated in the survey, so particular care must be taken when interpreting answers to questions that were specifically designed to gain insight into the views of these two particular groups. Although the results of this survey serve to highlight the general lack of awareness around TAND and the role and involvement of MHPs in the care of TSC patients, they also highlight that we currently know very little about the complex reasons and mechanisms underlying the TAND treatment gap. Another limitation is the heterogeneous definition of HCP specialty groups across different European countries, particularly the distinction between neurology and neuropsychiatry. Clinical neuropsychiatry is presently only regulated in three European countries [23], while training models in other European countries overlap with neurology and neuropsychology [24]. We sent the survey invitation to a list of prior attendees of a TSC convention for neurologists and neuropsychiatrists, and we were unable to report the numbers separately for each group based on the limited information that we received. Because the survey questionnaires were developed with four main HCP groups in mind (neurologists, psychiatrists, TSC specialists, HCPs with limited experience with TSC), we could only assume that neuropsychiatrists who responded to the survey used the questionnaire for neurologists. Finally, the survey could only investigate some aspects of our five hypotheses. Further research is necessary to study these mechanisms in more detail, with a focus on resources, multidisciplinary interaction, knowledge gaps, and stigmatization of mental disorders. In particular, our study highlights that it appears indispensable to investigate not only the views of patients, families, and caregivers, but also the views of psychiatrists and other MHPs about the treatment of TAND.

## Conclusions

This online survey confirms that there is a gap in awareness of TAND among TSC caregivers/families and HCPs alike. Consequently, the need for psychiatric and other MHP support appears to be often overlooked, as demonstrated by the minimal involvement of MHPs in the screening and long-term care of patients with TSC. Given that TSC is commonly diagnosed during childhood, TAND can have a significant impact on the patient’s developmental milestones and overall family dynamic. There is a need to raise awareness regarding TAND; to foster improvement of resources, professional knowledge, multidisciplinary interaction, and anti-stigmatization among HCPs regarding mental healthcare support to optimize the care of patients with TSC. In particular, more research, including that which considers the views of psychiatrists and other MHPs, is required to better understand the mechanisms underlying the TAND treatment gap.

## Supplementary Information

**Additional file 1.** Table S1: Survey questionnaire for TSC caregivers/families.

**Additional file 2.** Table S2: Survey questionnaire for TSC specialists.

**Additional file 3.** Table S3: Survey questionnaire for neurologists or child neurologists.

**Additional file 4.** Table S4: Survey questionnaire for psychiatrists or child psychiatrists.

**Additional file 5.** Table S5: Survey questionnaires for HCPs with limited experience with TSC.

**Additional file 6.** Fig. S1: Country of residence of HCPs.

**Additional file 7.** Fig. S2: Country of residence of TSC caregivers/families.

**Additional file 8.** Fig. S3: Usual investigations done by HCPs with limited experience with TSC.

**Additional file 9.** Fig. S4: Reasons for psychiatric referrals according to TSC specialists.

## Data Availability

Please contact the corresponding author for data requests.

## Abbreviations

EXPERT: EXchanging PERspectives on TSC
HCP: Healthcare provider
MHP: Mental health professional
TAND: TSC-associated neuropsychiatric disorder
TSC: Tuberous sclerosis complex
WHO: World Health Organization
