# Supplementary material for: Involvement of mental health professionals in the treatment of tuberous sclerosis complex–associated neuropsychiatric disorders (TAND): results of a multinational European electronic survey
Source: Orphanet J Rare Dis. 2021 May 12;16:216. doi: 10.1186/s13023-021-01800-w (PMC8117562; doi:10.1186/s13023-021-01800-w)
Supplement: Supplementary file 1 — Additional file 1. Table S1: Survey questionnaire for TSC caregivers/families. [file 13023_2021_1800_MOESM1_ESM.docx]

**Additional file 1: Table S1.** Survey questionnaire for TSC caregivers/families

| **Questions** | **Choices presented** | |
| --- | --- | --- |
| 1) Have you/the patient ever had a mental health/neurocognitive assessment, or filled out a TSC-Associated Neuropsychiatric Disorders checklist? | - No - Once (ever) - Once every 3 years | - Once a year - Twice a year - Every 3 months |
| 2) Do you feel that your/the patient’s psychiatric/neurocognitive requirements were appropriately managed by the physician? | - No - Somewhat | - Mostly - Yes |
| 3) Who/what raised the issue of TSC-Associated Neuropsychiatric Disorders (mental health or neurocognitive issues) with you/the patient? | - You/family member - Teacher - Rehabilitation professional - Physician | - TSC specialist - A screening process - Other (please specify) |
| 4) How easy is it for you/the patient to obtain a psychiatric assessment, if required? | - Easy - Difficult | - Very difficult - Not required |
| 5) How long have you/the patient been on a waiting list before being seen by a TSC specialist? | - 0 – 3 months - 3 – 6 months - 6 – 9 months | - 9 – 12 months - > 12 months |
| 6) How easy is it for you/the patient to obtain continuing psychiatric therapy, if required? | - Easy - Difficult | - Very difficult - Not required |
| 7) How effective do you consider the collaboration between your/the patient’s mental healthcare team, and the rest of your healthcare team to be? | - Ineffective - Somewhat effective - Mostly effective - Very effective | |
| 8) How does it feel for you/the patient to be referred for psychiatric assessment/treatment, compared with other healthcare assessment/treatment? | - The same - More negative - More positive | |
| 9) Answer (a) or (b) as applicable:   1. If you/the patient has not previously received psychiatric treatment for TSC: Do you think that psychiatric treatment could improve the quality of life for TSC patients and their families? 2. If you have/the patient has previously received psychiatric treatment for TSC: Do you think that psychiatric treatment can improve the quality of life for TSC patients and their families? | - Yes - No - Unsure | |

TSC, tuberous sclerosis complex
