# Supplementary material for: Involvement of mental health professionals in the treatment of tuberous sclerosis complex–associated neuropsychiatric disorders (TAND): results of a multinational European electronic survey
Source: Orphanet J Rare Dis. 2021 May 12;16:216. doi: 10.1186/s13023-021-01800-w (PMC8117562; doi:10.1186/s13023-021-01800-w)
Supplement: Supplementary file 2 — Additional file 2. Table S2: Survey questionnaire for TSC specialists. [file 13023_2021_1800_MOESM2_ESM.docx]

**Additional file 2: Table S2.** Survey questionnaire for TSC specialists^a^

| **Questions** | **Choices presented** | |
| --- | --- | --- |
| 1) How often do you refer your TSC patients to psychiatry? | - Never - Rarely - Sometimes | - Only if the patient/caregiver or HCP requests it - Once a year |
| 2) Which of the following TSC symptoms can be treated by the specialist doctors at your TSC clinic? | (Select all that are appropriate) | |
|  | - Seizures - Cognitive impairment | - Behavioral problems - Skin abnormalities |
| 3) For which of these symptoms would you refer a TSC patient to psychiatry? | (Select all that are appropriate)   - Learning disabilities - Aggression - Sudden rage - Attention Deficit Hyperactivity Disorder - Obsessive Compulsive Disorder - Autism - Self-harming behavior - Other (please specify) | |
| 4) In your experience, what are the barriers to effective collaboration between different doctor specialties and psychiatrists in terms of TSC treatment, if any? | (Select all that are appropriate)   - Lack of time/resources for multidisciplinary interactions - Culture - Lack of time/resources in psychiatry - Reluctance among psychiatrists to take on TSC patients because of a lack of knowledge/training in management of this rare and complex disease - Reluctance among patients to undergo psychiatric assessment/treatment in addition to multiple other diagnostic tests | |
| 5) How important do you think it is for psychiatrists to engage in TSC treatment of patients experiencing cognitive impairment or behavioral problems? | - Not important - Somewhat important - Important - Very important | |
| 6) How confident/comfortable do you feel in communicating the psychiatric assessment and treatment requirements and other psychiatric issues to a patient or patient family/caregiver? | - Not confident/comfortable - Somewhat confident/comfortable - Confident/Comfortable - Very confident/comfortable | |
| 7) In your experience, how often do you think that patients/patient’s families feel stigmatized when referred to psychiatric services? | - Never - Rarely - Sometimes | - Often - Always - I don’t know |
| 8) Do you think standard psychiatric therapy works for TSC patients? | - Never - Rarely - Sometimes | - Often - Always - I don’t know |

HCP, healthcare provider; TSC, tuberous sclerosis complex. ^a^TSC specialists are defined as those who are working in a TSC clinic
