# Supplementary material for: Involvement of mental health professionals in the treatment of tuberous sclerosis complex–associated neuropsychiatric disorders (TAND): results of a multinational European electronic survey
Source: Orphanet J Rare Dis. 2021 May 12;16:216. doi: 10.1186/s13023-021-01800-w (PMC8117562; doi:10.1186/s13023-021-01800-w)
Supplement: Supplementary file 3 — Additional file 3. Table S3: Survey questionnaire for neurologists or child neurologists. [file 13023_2021_1800_MOESM3_ESM.docx]

**Additional file 3: Table S3.** Survey questionnaire for neurologists or child neurologists

| **Questions** | **Choices presented** |
| --- | --- |
| 1) How regularly do you screen your patients for abnormal electric activity or seizure disorders? | - Never - Rarely - Sometimes - Only if the patient/caregiver or HCP/TSC specialist requests it - Once a year |
| 2) How are your patients screened? | - Medical interview - Checklist screening tool - Clinical exam in combination with CT - Clinical exam in combination with MRI - Other (please specify) |
| 3) In your experience, what are the barriers to effective assessment of neurocognitive difficulties in a TSC patient by HCPs? | - Lack of routine checks for neurocognitive symptoms known to be associated with TSC - Lack of neurophysiological tests - Limited experience with comprehensive assessment of cognitive development or behaviour - Limited experience with diagnostic criteria for psychiatric disorders - Other (please specify) |
| 4) How difficult is it for you to refer a TSC patient to psychiatry? | - Easy - Somewhat difficult - Difficult - Very difficult - Not required |
| 5) In your experience, what barriers are there to effective collaboration between neurologists and psychiatrists in terms of TSC treatment (if any)? | (Select all that are appropriate)   - Lack of time/resources for multidisciplinary interactions - Culture - Lack of time/resources in psychiatry - Lack of time/resources in neurology - Reluctance among psychiatrists to take on TSC patients because of a lack of knowledge/training in management of this rare and complex disease - Reluctance among neurologists to refer TSC patients for psychiatric assessment/treatment - Other (please specify) |
| 6) How confident are you in knowing when to refer a TSC patient to a psychiatrist? | - Not confident - Somewhat confident - Confident - Very confident |
| 7) How confident are you in discussing psychiatric assessment and treatment requirements with a TSC patient or patient family/caregiver? | - Not confident - Somewhat confident - Confident - Very confident |
| 8) In your experience, how often do you think that patients/patient’s families feel stigmatized when referred to psychiatric services? | - Never - Rarely - Sometimes - Often - Always - I don’t know |
| 9) Do you think standard psychiatric therapy works for TSC patients? | - Never - Rarely - Sometimes - Often - Always - I don’t know |

CT, computed tomography; HCP, healthcare provider; MRI, magnetic resonance imaging; TSC, tuberous sclerosis complex
