# Supplementary material for: Involvement of mental health professionals in the treatment of tuberous sclerosis complex–associated neuropsychiatric disorders (TAND): results of a multinational European electronic survey
Source: Orphanet J Rare Dis. 2021 May 12;16:216. doi: 10.1186/s13023-021-01800-w (PMC8117562; doi:10.1186/s13023-021-01800-w)
Supplement: Supplementary file 4 — Additional file 4. Table S4: Survey questionnaire for psychiatrists or child psychiatrists. [file 13023_2021_1800_MOESM4_ESM.docx]

**Additional file 4: Table S4.** Survey questionnaire for psychiatrists or child psychiatrists

| **Questions** | **Choices presented** |
| --- | --- |
| 1) Do you have resources available for TSC patients? | - No - Some - In most cases - Enough for every referral |
| 2) In your experience, what are the barriers to effective assessment of neurocognitive difficulties in a TSC patient by HCP? | - Lack of routine checks for neurocognitive symptoms known to be associated with TSC - Lack of neurophysiological tests - Limited experience with comprehensive assessment of cognitive development or behavior - Limited experience with diagnostic criteria for psychiatric disorders - Other (please specify) |
| 3) How often are TSC patients referred to you? | - Never - Rarely - Sometimes - Often - Very often |
| 4) How difficult is it for you to accept a new psychiatric TSC patient referral, which is not an emergency and at your discretion? | - Easy - Somewhat difficult - Difficult - Very difficult |
| 5) How many months is the average waiting list for non-emergency TSC referrals at your psychiatric hospital? | (open answer) |
| 6) In your experience, what barriers are there to effective collaboration between psychiatrists and non-psychiatric HCPs in terms of TSC treatment (if any)? | (Select all that are appropriate)   - Lack of time/resources for multidisciplinary interactions - Culture - Lack of time/resources in psychiatry - Reluctance among psychiatrists to take on TSC patients because of a lack of knowledge/training in management of this rare and complex disease - Reluctance among HCPs to refer TSC patients for psychiatric assessment/treatment - Other (please specify) |
| 7) How confident/comfortable do you feel with your standard psychiatric assessment/treatment tools when conducting them with a TSC patient? | - Not confident - Somewhat confident - Confident - Very confident |
| 8) How confident are you in discussing non-psychiatric healthcare requirements with a TSC patient or patient’s family/caregiver? | - Not confident - Somewhat confident - Confident - Very confident |
| 9) In your experience, how often do you think that patients/patient’s families feel stigmatized when referred to psychiatric services? | - Never - Rarely - Sometimes - Often - Always - I don’t know |
| 10) Do you think standard psychiatric therapy works for TSC patients? | - Never - Rarely - Sometimes - Often - Always - I don’t know |

HCP, healthcare provider; TSC, tuberous sclerosis complex
