# Supplementary material for: Involvement of mental health professionals in the treatment of tuberous sclerosis complex–associated neuropsychiatric disorders (TAND): results of a multinational European electronic survey
Source: Orphanet J Rare Dis. 2021 May 12;16:216. doi: 10.1186/s13023-021-01800-w (PMC8117562; doi:10.1186/s13023-021-01800-w)
Supplement: Supplementary file 5 — Additional file 5. Table S5: Survey questionnaires for HCPs with limited experience with TSC. [file 13023_2021_1800_MOESM5_ESM.docx]

**Additional file 5: Table S5.** Survey questionnaires for HCPs with limited experience with TSC^a^

| **Questions** | **Choices presented** | |
| --- | --- | --- |
| 1) Which of these tests do you regularly perform to diagnose TSC? | - MRI of the brain - CT of the lungs, liver and kidneys - Ultrasound scan of the kidneys | - Echocardiogram - Ultrasound eye exam - Skin exam - Genetic testing - Other (please specify) |
| 2) In your experience, what are the barriers to effective assessment of neurocognitive difficulties in a TSC patient? | - Lack of routine checks for neurocognitive symptoms known to be associated with TSC - Lack of neurophysiological tests - Limited experience with comprehensive assessment of cognitive development or behavior - Limited experience with diagnostic criteria for psychiatric disorders - Other (please specify) | |
| 3) How often do you assess cognitive and behavioral issues in TSC? | - Never - Rarely - Sometimes - Only if the patient/caregiver or HCP requests it - Once a year | |
| 4) How difficult is it for you to refer a patient to psychiatry? | - Easy - Somewhat difficult - Difficult | - Very difficult - Not required |
| 5) In your experience, what barriers are there to effective collaboration between non-psychiatric HCPs and psychiatrists in terms of TSC treatment, if any? | (Select all that are appropriate)   - Lack of time/resources in psychiatry - Reluctance among psychiatrists to take on TSC patients due to the multifaceted nature of the disease/lack of disease understanding - Reluctance among HCPs to refer TSC patients for psychiatric assessment/treatment - Other (please specify) | |
| 6) How confident are you in knowing when to refer a patient to a psychiatrist? | - Not confident - Somewhat confident | - Confident - Very confident |
| 7) How often did you refer a patient to a psychiatrist? | - Never - Rarely - Sometimes | - Regularly - Always |
| 8) How confident are you in discussing psychiatric assessment and treatment requirements with a patient or patient family/caregiver? | - Not confident - Somewhat confident - Confident - Very confident | |
| 9) In your experience, how often do you think that patients/patient’s families feel stigmatized when referred to psychiatric services? | - Never - Rarely - Sometimes | - Often - Always - I don’t know |
| 10) In your experience, do you think standard psychiatric therapy works for TSC patients? | - Never - Rarely - Sometimes | - Often - Always - I don’t know |

- CT, computed tomography; HCP, healthcare provider; MRI, magnetic resonance imaging; TSC, tuberous sclerosis complex. ^a^HCPs with limited experience with TSC refer mainly to primary care physicians
