# Supplementary material for: Involvement of mental health professionals in the treatment of tuberous sclerosis complex–associated neuropsychiatric disorders (TAND): results of a multinational European electronic survey
Source: Orphanet J Rare Dis. 2021 May 12;16:216. doi: 10.1186/s13023-021-01800-w (PMC8117562; doi:10.1186/s13023-021-01800-w)
Supplement: Supplementary file 7 — Additional file 7. Fig. S2: Country of residence of TSC caregivers/families. [file 13023_2021_1800_MOESM7_ESM.docx]

**Fig. S2** Country of residence of TSC caregivers/families.
