# Supplementary material for: Involvement of mental health professionals in the treatment of tuberous sclerosis complex–associated neuropsychiatric disorders (TAND): results of a multinational European electronic survey
Source: Orphanet J Rare Dis. 2021 May 12;16:216. doi: 10.1186/s13023-021-01800-w (PMC8117562; doi:10.1186/s13023-021-01800-w)
Supplement: Supplementary file 8 — Additional file 8. Fig. S3: Usual investigations done by HCPs with limited experience with TSC. [file 13023_2021_1800_MOESM8_ESM.docx]

-
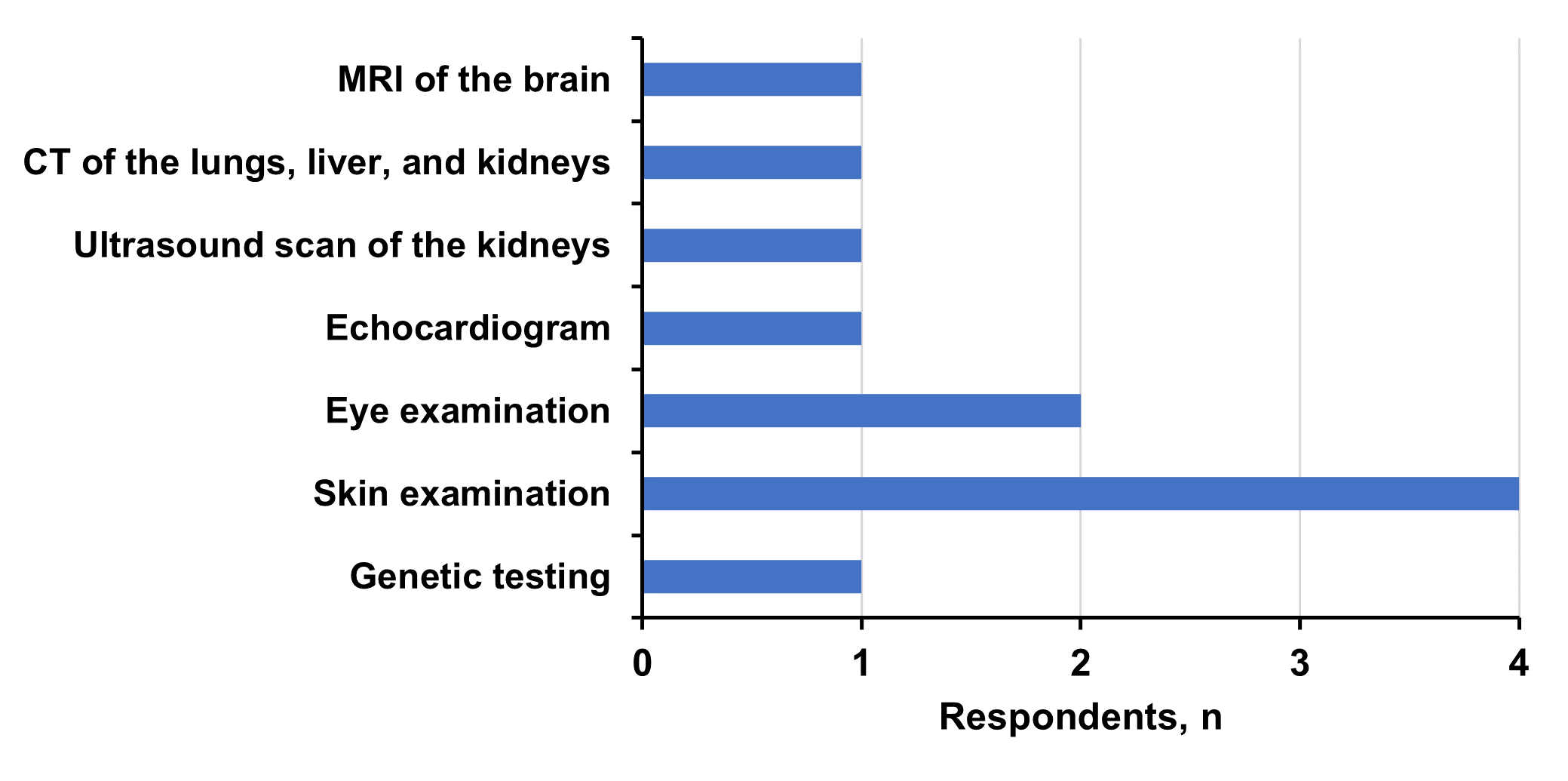


**Fig. S3** Usual investigations done by HCPs with limited experience with TSC.^a^ CT, computed tomography; HCPs, healthcare providers; MRI, magnetic resonance imaging; TSC, tuberous sclerosis complex. ^a^HCPs with limited experience with TSC refer mainly to primary care physicians.
