# Supplementary material for: Involvement of mental health professionals in the treatment of tuberous sclerosis complex–associated neuropsychiatric disorders (TAND): results of a multinational European electronic survey
Source: Orphanet J Rare Dis. 2021 May 12;16:216. doi: 10.1186/s13023-021-01800-w (PMC8117562; doi:10.1186/s13023-021-01800-w)
Supplement: Supplementary file 9 — Additional file 9. Fig. S4: Reasons for psychiatric referrals according to TSC specialists. [file 13023_2021_1800_MOESM9_ESM.docx]

-
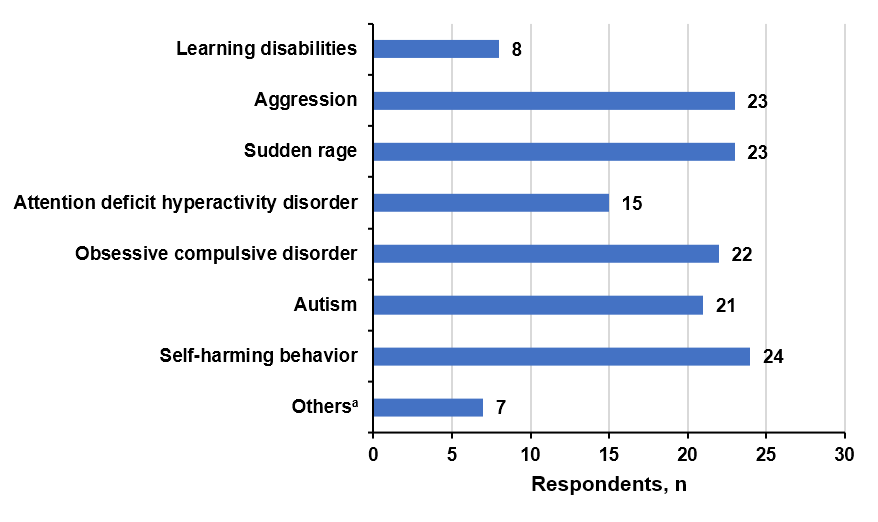

- **Fig. S4** Reasons for psychiatric referrals according to TSC specialists. TSC, tuberous sclerosis complex. ^a^Answers supplied by respondents under the “Others” field were: (1) Disorders not explained by a somatic disorder and not responding or insufficiently responding to first-line drug treatment, or a situation immediately appearing to be psychiatric and severe (delirium, hallucinations, catatonia, endangerment); (2) Any other serious psychological or psychiatric problem (e.g., depression or anxiety) that is not responding to simple measures such as advice or drugs that can be prescribed by general physicians; (3) Sleep disorder; (4) Failure to respond to initial management by community pediatrician or patient over 18 years of age; (5) Sexual harassment; (6) Adult patients with depression and anxiety.
